# PICK1-Deficient Mice Exhibit Impaired Response to Cocaine and Dysregulated Dopamine Homeostasis

**DOI:** 10.1523/ENEURO.0422-17.2018

**Published:** 2018-06-11

**Authors:** Kathrine Louise Jensen, Gunnar Sørensen, Ditte Dencker, William Anthony Owens, Troels Rahbek-Clemmensen, Michael Brett Lever, Annika H. Runegaard, Nikolaj Riis Christensen, Pia Weikop, Gitta Wörtwein, Anders Fink-Jensen, Kenneth L. Madsen, Lynette Daws, Ulrik Gether, Mattias Rickhag

**Affiliations:** 1 Molecular Neuropharmacology and Genetics Laboratory, Department of Neuroscience, Faculty of Health and Medical Sciences, University of Copenhagen, Copenhagen DK-2200, Denmark; 2 Laboratory of Neuropsychiatry, Psychiatric Center Copenhagen, Faculty of Health and Medical Sciences, University of Copenhagen, Copenhagen DK-2200, Denmark; 3 Department of Cellular and Integrative Physiology, University of Texas Health Science Center at San Antonio, TX 78229

**Keywords:** cocaine, dopamine homeostasis, drug addiction, protein interacting with C-kinase 1, striatum, tyrosine hydroxylase

## Abstract

Protein interacting with C-kinase 1 (PICK1) is a widely expressed scaffold protein known to interact via its PSD-95/discs-large/ZO-1 (PDZ)-domain with several membrane proteins including the dopamine (DA) transporter (DAT), the primary target for cocaine’s reinforcing actions. Here, we establish the importance of PICK1 for behavioral effects observed after both acute and repeated administration of cocaine. In PICK1 knock-out (KO) mice, the acute locomotor response to a single injection of cocaine was markedly attenuated. Moreover, in support of a role for PICK1 in neuroadaptive changes induced by cocaine, we observed diminished cocaine intake in a self-administration paradigm. Reduced behavioral effects of cocaine were not associated with decreased striatal DAT distribution and most likely not caused by the ∼30% reduction in synaptosomal DA uptake observed in PICK1 KO mice. The PICK1 KO mice demonstrated preserved behavioral responses to DA receptor agonists supporting intact downstream DA receptor signaling. Unexpectedly, we found a prominent increase in striatal DA content and levels of striatal tyrosine hydroxylase (TH) in PICK1 KO mice. Chronoamperometric recordings showed enhanced DA release in PICK1 KO mice, consistent with increased striatal DA pools. Viral-mediated knock-down (KD) of PICK1 in cultured dopaminergic neurons increased TH expression, supporting a direct cellular effect of PICK1. In summary, in addition to demonstrating a key role of PICK1 in mediating behavioral effects of cocaine, our data reveal a so far unappreciated role of PICK1 in DA homeostasis that possibly involves negative regulation of striatal TH levels.

## Significance Statement

Cocaine addiction is a major societal problem and better treatments are needed. We demonstrate here the importance of the PSD-95/discs-large/ZO-1 (PDZ)-domain scaffold protein protein interacting with C-kinase 1 (PICK1) for both the acute reinforcing effects of cocaine and the long-term behavioral changes seen as a consequence of repeated cocaine exposure. Interestingly, our data suggest that these alterations are independent of PICK1 binding to the primary target of cocaine, the dopamine transporter (DAT). Moreover, our study reveals a novel role of PICK1 in maintenance of DA homeostasis that involves negative regulation of the levels of striatal tyrosine hydroxylase (TH), the rate-limiting enzyme in DA synthesis. Summarized, these data provide an important framework for further exploring the role of PICK1 in addiction and as putative target for treatment of psychostimulant abuse.

## Introduction

Cocaine is a highly addictive and widely abused psychostimulant (Karila et al., 2012; Proctor, 2012; Degenhardt et al., 2013; Karila et al., 2014). Treatment of cocaine abuse, however, remains a major challenge and still no pharmacological agents have proven therapeutically useful (Kristensen et al., 2011; Shorter et al., 2015). The reinforcing properties of cocaine can be attributed primarily to its high-affinity interaction with the presynaptic plasma membrane dopamine (DA) transporter (DAT). DAT mediates rapid reuptake of DA from the extracellular space (Giros et al., 1996; Kristensen et al., 2011) and is thereby critical for replenishment of intracellular striatal DA pools (Jones et al., 1998). Binding of cocaine to DAT leads to a fast increase in extracellular DA, stimulation of DA receptors and subsequent long-lasting changes in synaptic plasticity, which are thought to underlie the craving for continuous drug intake (Chen et al., 2006; Thomsen et al., 2009).

Protein interacting with C-kinase 1 (PICK1) is a scaffold protein implicated in several cellular functions (Xu and Xia, 2006; Hanley, 2008; Focant and Hermans, 2013). Scaffold proteins constitute a highly diverse family of proteins that serve a key role in orchestrating neuronal signaling processes ensuring specificity in intracellular signaling networks (Kim and Sheng, 2004; Good et al., 2011). PICK1 is unique by possessing both a PSD-95/discs-large/ZO-1 (PDZ) protein interaction domain and a lipid binding BAR (Bin/amphiphysin/Rvs) domain (Hanley, 2008; Madsen et al., 2012). The BAR-domain is dimeric and thus PICK1 contains in its functional form two separate PDZ-domains allowing simultaneous binding of two interaction partners (Xu and Xia, 2006; Hanley, 2008; Focant and Hermans, 2013). The PDZ-domain of PICK1 interacts with the C termini of several receptors, channels and transporters expressed in the CNS (Xia et al., 1999; Dev et al., 2000; Torres et al., 2001; Madsen et al., 2005). These include DAT (Torres et al., 2001; Bjerggaard et al., 2004) and the AMPA receptor (AMPAR) subunit ionotropic glutamate receptor 2 (GluA2; Xia et al., 2000; Kim et al., 2001; Jin et al., 2006; Steinberg et al., 2006; Thorsen et al., 2010), and for both, it has been suggested that PICK1 modulates their trafficking (Xia et al., 2000; Kim et al., 2001; Torres et al., 2001; Jin et al., 2006; Steinberg et al., 2006; Thorsen et al., 2010). Interfering with the interaction between PICK1 and AMPA receptors has previously been shown to blunt the synaptic plasticity induced at glutamatergic synapses onto ventral tegmental area (VTA) dopaminergic neurons on a single cocaine exposure (Bellone and Lüscher, 2006). Moreover, both cerebellar and hippocampal long-term depression is abolished on disruption of the PICK1-GluA2 interaction (Xia et al., 2000; Kim et al., 2001; Jin et al., 2006; Steinberg et al., 2006; Thorsen et al., 2010). However, the consequences of disrupting PICK1-DAT interactions for the behavioral effects of cocaine are unknown.

Here, we investigate the importance of PICK1 for the behavioral actions of cocaine. In mice with targeted deletion of PICK1 [PICK1 knock-out (KO) mice], the acute locomotor response to a single injection of cocaine was markedly attenuated. Moreover, cocaine intake was diminished in a self-administration paradigm. These behavioral changes were not associated with altered presynaptic DAT expression or aberrant postsynaptic DA receptor activation. Intriguingly, we found that PICK1 KO mice displayed increased striatal levels of tyrosine hydroxylase (TH) and DA, relative to wild-type littermates. Chronoamperometric recordings in striatum revealed enhanced DA release in PICK1 KO mice further supporting increased striatal DA pools. The importance of PICK1 specifically in DA neurons was confirmed by lentiviral knock-down (KD) of PICK1 in cultured midbrain dopaminergic neurons resulting in elevated TH expression. However, neither coimmunoprecipitation nor fluorescence polarization experiments showed a direct interaction between TH and PICK1. Our data demonstrate the importance of PICK1 for regulating behavioral effects of cocaine, and reveal a so far unknown role of PICK1 in maintenance of DA homeostasis.

## Materials and Methods

### Subjects

Male PICK1 KO mice (Gardner et al., 2005), DAT + Ala knock-in mice (Rickhag et al., 2013) and wild-type littermates of at least 10 weeks of age at the beginning of an experiment were used, unless otherwise specified. Mice were group-housed in a temperature-controlled room maintained on a 12/12 h light/dark cycle (lights on at 7 A.M.) with access to standard rodent chow and water *ad libitum*. Animals were allowed to habituate to the facility for at least 7 d before initiation of experiments. Animal experiments were performed in accordance with the guidelines of the Danish Animal Experimentation Inspectorate (permission number 2012-15-293-00279 and 2012-15-2934-00038) in a fully AAALAC (American Association for Laboratory Animal Care)-accredited facility under the supervision of a local animal welfare committee. Chronoamperometric experiments conducted at University of Texas Health Science Center at San Antonio were performed in accordance with Institutional Animal Care and Use Committee approved protocols, in a fully AAALAC-accredited facility. All efforts were made to minimize pain and discomfort as well as the number of animals used during the experiments.

### Drugs

SKF82958 hydrobromide and quinpirole hydrochloride was purchased from Sigma-Aldrich. Cocaine hydrochloride was obtained from the Copenhagen University Hospital Pharmacy. All drugs were prepared in 0.9% aqueous NaCl, with a final pH 6–7 and injected intraperitoneally in a volume of 10 ml/kg.

### Drug-induced hyperlocomotion without habituation

Locomotor activity was measured in activity boxes as previously described (Sørensen et al., 2012; Rickhag et al., 2013). Mice (wild-type/PICK1 KO/DAT + Ala mice) were injected with either 0-, 5-, 10-, or 30-mg/kg cocaine dissolved in saline intraperitoneally 3–7 min before testing. The animals were then placed in a cage and the activity was measured for 1 h. Each mouse only received one dose of cocaine and cages were cleaned between tests.

### Drug-induced hyperlocomotion with habituation

PICK1 KO and wild-type mice were habituated to the experimental room for a minimum of 30 min before the experiment. The mice were placed in the open field (40 × 40 × 80 cm) for a 120-min habituation period followed by intraperitoneal administration of the DA D1 receptor (D_1_R) agonist, SKF82958 (0.3 or 1 mg/kg), cocaine (10 or 30 mg/kg), DA D2 receptor (D_2_R) agonist, quinpirole (0.1 or 10 mg/kg), or vehicle. With a minimum 7-d washout period, mice were retested in a counterbalanced Latin-square design so that each animal only received the same drug once. A video camera placed above the open field recorded the animals’ behavior and the distance traveled by the animal was analyzed using the video-tracking software EthoVision (Noldus). For best possible comparison to the effect of cocaine on the unhabituated mice, locomotion was analyzed for the 60 min before and after drug injection (injection at *t* = 120).

### Cocaine-induced locomotor sensitization

After administration of saline on the initial day (day 0), PICK1 KO and wild-type mice were divided into two groups that received either cocaine (10 mg/kg, i.p.) or saline for 6 d (day 1–6). All mice were given cocaine (10 mg/kg, i.p.) on day 12 and day 20 and retested in the open field. On day 21, all mice were injected with saline and re-exposed to the open field to examine whether context conditioning had any effect on the observed response. Mice spent 60 min in the open field box on all testing days, and locomotion was recorded and analyzed using EthoVision (Noldus).

### Intravenous self-administration of cocaine or liquid food

Equipment, training, and evaluation procedures were performed as previously described for chronic self-administration of cocaine or liquid food (Schmidt et al., 2011; Sørensen et al., 2015).

Cocaine (1.0 mg/kg per infusion) was available under a fixed ratio 1:1 (FR1) schedule in daily 3-h sessions, 5–6 d per week, until baseline criteria was met (≥20 reinforcers earned, with ≤20% variation over two consecutive sessions and ≥70% responses in the active hole). In consecutive sessions, saline was substituted for cocaine until extinction criteria were met (<80% of the baseline responding for cocaine self-administration). This was followed by reintroduction of the training dose until previously established baseline criteria were met again or a new baseline was established with similar requirements as above. Subsequently, dose-effect functions (saline, 0.03, 0.1, 0.3, 1.0 mg/kg per infusion of cocaine) were determined for each mouse according to a Latin-square design twice in each mouse. To prevent overdosing, total drug intake was limited to 30 mg/kg per session.

Another set of experimentally naïve mice was used for self-administration of a nondrug reinforcer under a FR1 schedule. The mice were food deprived for 18–20 h (with *ad libitum* water) before the first presentation of liquid food (5 ml of Nutridrink, vanilla flavor, Nutricia) in the operant chamber. When ≥1.5 ml of the 5 ml available was consumed per 2-h session, mice were placed in the operant chamber with one active and one inactive nose-poke hole for daily 2-h sessions similar to cocaine FR1 self-administration. Acquisition lasted until criteria were met (≥20 reinforcers earned, with ≤20% variation over two consecutive sessions and ≥70% responses in the active hole). Subsequently, water was substituted until responding was extinguished to <80% of food-maintained responding. This was followed by reintroduction of the training concentration of liquid food until previously established baseline criteria were met again or a new baseline was established with similar requirements as above. Then, a range of liquid food dilutions (Nutridrink: water; 3%, 10%, 32%, and 100%) was presented according to a Latin-square design, determined twice in each mouse.

### Immunoblotting

Equal amounts of samples, prepared as described in the following sections, were run on a 10/15 well any kDa, prestacked gel (Bio-Rad) and transferred to PVDF membranes (Millipore). Membranes were blocked in either 5% bovine serum albumin in 0.05% PBS/Tween 20 or 2% PVP-40 in 0.05% PBS/Tween 20 (phospho-proteins) or in 5% dry milk in 0.05% PBS/Tween 20 (unphoshorylated proteins), and incubated overnight with antibodies against flotillin (1:1000, SAB2500404, rabbit polyclonal, Sigma), DAT (1:1000, MAB369, rat monoclonal, Millipore), D_1_R (1:1000, D2944, rat polyclonal, Sigma), TH (1:1000, MAB318, mouse monoclonal, Millipore), phospho-TH (1:1000, 2791S, rabbit polyclonal, Cell Signaling), cAMP response element binding protein (CREB; 1:1000, 48H2, rabbit polyclonal, Cell Signaling), anti-phospho-Ser133 CREB (1:1000, p1010-133, PhospoSolutions), or vesicular monoamine transporter-2 (VMAT2; 1:1000, Ab191121, rabbit polyclonal, Abcam) depending on the experiment. Following incubation with horseradish peroxidase (HRP)-conjugated anti-rabbit/anti-mouse/anti-rat antibodies (1:2000), the blots were visualized by chemiluminescence (ECL kit, GE Healthcare for un-phosphorylated proteins and SuperSignal ELISA Femto Maximum Sensitivity Substrate, Thermo Scientific for phosphorylated proteins) using AlphaEase (Alpha Innotech). To verify equal protein loading, the membranes were probed with antibodies against Na^+^/K^+^ ATPase (1:1000, Ab7671, mouse monoclonal, Abcam) or HRP-conjugated β-actin (1:10000, A3854, mouse monoclonal, Sigma). In surface biotinylation experiments, band intensities of biotinylated DAT and D_1_R were normalized to the band intensity of total DAT and D_1_R in the respective input lysates.

### Preparation of striatal lysates for surface biotinylation experiments

Surface biotinylation was performed as previously described (Runegaard et al., 2017).

### Preparation of striatal and midbrain lysates for immunoblotting

Adult mice were killed by decapitation and brains rapidly removed. Striatum and midbrain were rapidly dissected and homogenized in RIPA lysis buffer (1% Triton X-100, 1 mM EGTA, 1 mM EDTA, 150 mM NaCl, 1% NP-40, 10 mM TRIS-HCl; pH 7.4) supplemented with protease inhibitor cocktail (Roche Diagnostics) and phosphatase inhibitor cocktail 3 (Sigma Aldrich). Tissue was incubated on ice for 20 min followed by centrifugation at 16,000 × *g* for 20 min at 4°C. Supernatant was transferred to new tubes and an aliquot was removed to determine protein concentration using a BCA Protein Assay kit (Pierce). Samples were eluted in SDS loading buffer and left at 37°C for 30 min followed by immunoblotting.

### Preparation of striatal lysates for sucrose gradient centrifugation experiments

Adult mice were killed by decapitation and brains rapidly removed. Striatum from PICK1 wild-type and KO mice were rapidly dissected and homogenized in homogenization buffer (0.32 M sucrose and 4 mM HEPES; pH 7.4). Samples were centrifuged at 1000 × *g* for 10 min at 4°C to remove cell nuclei followed by centrifugation at 16,000 × *g* for 20 min at 4°C to pellet the membranes. The pellet was homogenized in 1-ml lysis buffer [1% Brij56 (w/V)], protease inhibitor, and 0.2 mM PMSF in the gradient buffer (25 mM HEPES and 150 mM NaCl; pH 7.4) for 15 min on ice. A 1-ml sample was mixed with 1-ml sucrose (80% w/V) in gradient buffer to create 2 ml with final 40% (w/V) sucrose content in a 15-ml centrifuge tube (Beckman Coulter). On top of this, a 12 ml 35–15% continuous gradient was assembled using a SG15 gradient maker (Hoefer). Subsequently the gradient was ultracentrifuged at 100,000 × *g* for 18 h in a Beckman SW28.1 rotor head (Beckman Coulter) and fractionated into nine fractions using a P1 pump. Each fraction was incubated for 30 min at 37°C in SDS loading buffer with a final concentration of 66 mM DTT followed by immunoblotting.

### Preparation of striatal and midbrain lysates for coimmunoprecipitation experiments

Adult male C57BL/6 mice (age, two to three months) were killed and brains immediately placed in ice-cold artificial CSF (aCSF; 124 mM NaCl, 3 mM KCl, 26 mM NaHCO_3_, 1.25 mM NaH_2_PO_4_, 2 mM CaCl_2_, 1 mM MgSO_4_, and 10 mM D-glucose) while striatum and midbrain were dissected. Tissue was homogenized in lysis buffer [50 mM Tris (pH 7.4), 150 mM NaCl, 0.1% SDS, 0.5% Na deoxycholate, 1% Triton X-100, 5 mM NaF, and 1× Roche protease inhibitor cocktail]. Lysates were centrifuged at 20,000 × *g* for 15 min and supernatants precleared with streptavidin beads (Dynabeads MyOne Streptavidin T1, Thermo). A total of 500-μg protein lysate was incubated with 5-μg TH/PICK1 Ab (TH, MAB318 Millipore; PICK1 rabbit, a kind gift from Dr. Richard Huganir, Johns Hopkins University, Baltimore, MD) and incubated at 4°C, rotating overnight. Protein-G beads were added and samples left for an additional 3 h. Beads were washed in lysis buffer and proteins were eluted in SDS loading buffer and left at 100°C for 6 min followed by immunoblotting.

### Striatal DA uptake

Synaptosomal uptake assay was performed as described previously (Jensen et al., 2017; Runegaard et al., 2017).

### HPLC determination of striatal DA content

HPLC analysis was performed as described previously (Jensen et al., 2017).

### *In viv*o high-speed chronoamperometry

*In vivo* high-speed chronoamperometry was conducted using the FAST-12 system (Quanteon; http://www.quanteon.cc) as previously described (Owens et al., 2005; Williams et al., 2007; Daws et al., 2016) with some modification. Recording electrode/micropipette assemblies were constructed using a single carbon-fiber (30 μm in diameter; Specialty Materials), which was sealed inside fused silica tubing (SCHOTT North America). The exposed tip of the carbon fiber (150 μm in length) was coated with 5% Nafion (Aldrich Chemical Co; three to four coats baked at 200°C for 5 min per coat) to provide a 1000-fold selectivity of DA over its metabolite 3,4-dihydroxyphenylacetic acid (DOPAC). Under these conditions, microelectrodes display linear amperometric responses to 0.25–10 μM DA during *in vitro* calibration in 100 mM PBS (pH 7.4).

Male wild-type or PICK1 KO mice weighing 26–36 g were anesthetized by intraperitoneal injection (10-ml/kg body weight) of a mixture of urethane (70 mg/ml) and α-chloralose (7 mg/ml), then fitted with an endotracheal tube to facilitate breathing, and placed into a stereotaxic frame (David Kopf Instruments). Body temperature was maintained by placing the animals on a water-circulated heating pad. To locally deliver potassium chloride (KCl; 200 µM, Sigma, pH 7.4) close to the recording site, a glass multi-barrel micropipette (FHC) was positioned adjacent to the microelectrode using sticky wax (Moyco). The center-to-center distance between the microelectrode and the micropipette ejector was ∼200 μm. The electrode/micropipette assembly was lowered into the striatum at the following coordinates (in mm from bregma; Franklin, 1997): A/P, +1.1; M/L, ±1.4; and D/V, −2.25. To evoke release of endogenous DA, KCl was pressure-ejected using a Picospritzer II (General Valve Corporation) in an ejection volume 43 ± 7 nl, 8.5 ± 1 pmol and 41 ± 6 nl, 8.0 ± 1 pmol, for wild type and KO, respectively. To record KCl-evoked efflux of DA at the carbon fiber electrode, oxidation potentials consisting of 100-ms pulses of 550 mV, each separated by a 900-ms interval, during which the resting potential was maintained at 0 mV, were applied with respect to an Ag/AgCl reference electrode implanted into the contralateral superficial cortex. Oxidation and reduction currents were digitally integrated during the last 80 ms of each 100-ms voltage pulse. For each recording session, DA was identified by its reduction/oxidation current ratio, which ranged from 0.50 to 0.83. Detailed methods can be found in Daws et al. (2016). Peak signal amplitude was used as an index of striatal tissue pools of DA. At the conclusion of each experiment, an electrolytic lesion was made to mark the placement of the recording electrode tip. Mice were then decapitated while still anesthetized, and their brains were removed, frozen on dry ice, and stored at −80°C until sectioned (20 μm) for histologic verification of electrode location within the striatum.

### Isolation and mRNA expression analysis

Adult mice were killed by decapitation and brains rapidly removed. Midbrain from PICK1 wild-type and KO mice was rapidly dissected in the presence of RNase away (Molecular Bioproducts, Fischer scientific). Samples were collected on dry ice and placed at -80˚C until RNA extraction. Samples were lyzed using a QIAGEN microRNA column (QIAGEN) according to the manufacturer’s instructions, including DNase treatment. Conversion from RNA to cDNA was done with SuperScript III (Thermo Fisher). Reaction 1 (70°C for 5 min): max 1-µg RNA, 50-ng random primer, 0.2 µmol DTT, H_2_O to a final volume 15 µl. Reaction 2 (10 min at 25°C, 50 min at 50°C, and 5 min at 80°C): 10× reverse transcription buffer, 10 nmol dNTP mix, 40-U RNaseOUT, 200-U superscript, H_2_O to a final volume of 5 µl, thereafter added to reaction 1. Before running qPCR on the samples, cDNA was diluted 10× with sterile water. The Agilent Mx3000p (Agilent Technologies) real-time thermocycler was used to perform the qPCR using SYBR green (PrecisionPLus 2× qPCR Mastermix Primer design Ltd.) as probe. Data collection was performed by MxPro software and analyzed in Microsoft Excel as well as GraphPad Prism version 6. All samples were run in duplicates and β-actin was used as reference/housekeeping gene. Relative expression was calculated through the ΔΔC_q_ method (Pfaffl, 2001). Primers for β-actin forward: TTCTACAATGAGCTGCGTGTG and reverse: GGGGTGTTGAAGGTCTCAAA; TH forward: CCGTCATGCCTCCTCACCTATG and reverse: CCTGGGAGAACTGGGCAAATG.

### Protein expression, purification, and fluorescence polarization assay

*Escherichia coli* transformed with a PICK1 encoding plasmid (pET41; Madsen et al., 2005) were inoculated overnight in 50 ml of lysogeny broth media (+kanamycin), diluted into 1-l lysogeny broth media (+kanamycin) and grown at 37°C to OD 0.6. Protein expression was induced with 0.5 mM IPTG and grown overnight at 20°C. Cells were harvested and resuspended in lysis buffer containing 50 mM Tris, 125 mM NaCl, 2 mM DTT (Sigma), 1% Triton X-100 (Sigma), 20-µg/ml DNase 1, and cOmplete protease inhibitor cocktail (Roche). Lysate was frozen at -80°C to induce cell lysis. The bacterial suspension was thawed and cleared by centrifugation (F20 rotor, 36,000 × *g* for 30 min at 4°C). Supernatant was incubated with glutathione-sepharose 4B beads (GE Healthcare) for 2 h at 4°C under gentle rotation. The beads were pelleted at 4000 × *g* for 5 min and washed twice in 50 mM Tris, 125 mM NaCl, 2 mM DTT, and 0.01% Triton X-100. Beads were transferred to a PD10 gravity column and were washed three times. Protein was separated from GST by thrombin (Millipore) incubation overnight at 4°C with gentle rotation. PICK1 was eluted on ice and absorption at 280 nm was measured on a TECAN plate reader, followed by protein concentration calculations using lambert beers law (A = εcl), εA280PICK1 = 32320(cm*mol/l)-1. A peptide of the 11 most C-terminal residues of mouse TH (HTLTQALSAIS) was ordered from TAGcopenhagen A/S with >95% purity. The peptide was dissolved in 10% DMSO and wash buffer to a concentration of 2 mM, and a final assay max concentration of 1 mM was used. The fluorescence polarization assay was performed as previously described (Madsen et al., 2005; Erlendsson et al., 2014) with a fixed concentration of PICK1 (1 µM, nonsaturated) and fluorescent tracer (Oregon-Green DATC13, 20 nM), preincubated for 15 min after which increasing concentrations of unlabeled TH-C11 peptide were added and incubated for 20 min on ice in a black 190-µl nonbinding surface 96-well plate (Corning). Fluorescence polarization was measured on an Omega POLARstar plate reader, 488-nm excitation and 535-nm emission.

### Confocal microscopy

Confocal microscopy was performed using a Zeiss LSM 510 confocal laser-scanning microscope with an oil immersion 63 × 1.4 numerical aperture objective (Carl Zeiss). Alexa Fluor 488 dye was excited with a 488-nm laserline from an argon-krypton laser and emitted light detected using a 505- to 530-nm bandpass filter. Alexa Fluor 568 dye was excited with a 543-nm helium-neon laser, and fluorescence was recorded using a 560-nm long-pass filter. Images were analyzed with ImageJ (FIJI) software.

### Immunohistochemistry of perfused brain sections for confocal microscopy

Adult mice were anaesthetized and transcardially perfused with 4% paraformaldehyde in 0.1 M PBS. Coronal midbrain sections (40 μm) were used for fluorescence immunohistochemistry. Initially, antigen retrieval was performed by treatment with 10 mM sodium citrate buffer (pH 6.0) for 30 min at 80°C. Brain sections were then rinsed in PBS and preincubated in PBS containing 5% goat serum, 1% bovine serum albumin and 0.3% Triton X-100 for 1 h. Sections were subsequently incubated with a custom generated mouse monoclonal PICK1 antibody (1:1000, 2G10; described in Jansen et al., 2009) and rabbit polyclonal TH (1:1000, OPA-04050, Affinity Bioreagents) at 4°C overnight. Sections were rinsed in washing buffer (0.25% BSA, 0.1% Triton X-100 in PBS) and then incubated with biotinylated goat anti-mouse (1:400, E0433, DAKO Cytomation A/S) or Alexa Fluor 568 goat anti-rabbit IgG (1:1000, A11036, Invitrogen). Following rinsing, slices were incubated with avidin-biotin-peroxidase complex (Vector Laboratories), rinsed, and incubated with biotinyl tyramide amplification reagent (PerkinElmer) and incubated with streptavidin-conjugated Alexa Fluor 488 (1:400, S11223, Thermo Fisher Scientific) to detect PICK1. Additional rinsing was followed by mounting on SuperFrost slides (Menzel-Gläser, Braunschweig, Germany) and cover-slipping using Prolong Gold antifade reagent (Invitrogen).

### Culturing, transduction, and immunocytochemistry of dopaminergic neurons for confocal microscopy

Dopaminergic neurons were isolated from ventral midbrain tissue dissected from 1- to 3-d-old rat pups and plated on a monolayer of glia cells on coverslips using a protocol modified from (Rayport et al., 1992) as described in (Eriksen et al., 2009). For lentiviral transduction of the neurons, vectors were driven by the dual promoter FUGW with the H1 promoter driving the expression of the small hairpin (ShPICK1) targeting the PICK1 sequence (CTATGAGTACCGCCTTATCCT) and the ubiquitin promoter driving the expression of GFP (PICK1 KD; Citri et al., 2010). In the control vector (GFP), the sequence of the ShPICK1 was removed before the PCR product of the deleted ShPICK1 fragment and the PICK1 KD vector was digested and ligated together using AfeI and BamHI. Removal of ShPICK1 was achieved by synthesis of a 500 bp PCR fragment using the primers AGTAACGGATCCTTTTTCTAGCCCCAAGGGCG and TCGCCGAGAAGGGACTACTTTTCCTCGCCTG and the original PICK1 KD construct as template. Consequently, the control GFP vector only drives the expression of GFP under the ubiquitin promoter. Verification of both constructs was performed by dideoxynucleotide sequencing (Eurofins Genomics, Ebersberg, Germany). Transduction of dopaminergic neurons was initiated the first day after plating dopaminergic neurons on the monolayer of glia cells. Before transduction, half the medium was removed from each well and kept in incubator. Equal amounts of virus inducing expression of either PICK1 KD or GFP were added below the medium surface in two to three spots. After 6–8 h, the media was transferred back to the dopaminergic neurons and 5-fluorodeoxyuridine was added to the medium to inhibit cell growth. For immunocytochemistry, dopaminergic neurons were washed followed by 15-min fixation in 4% paraformaldehyde. Cells were washed followed by 20 min in permeabilization + blocking buffer (0.2% saponin + 5% goat serum in PBS). Neurons were stained with a rabbit anti-TH antibody (1:1000, OPA1-04050, Thermo Fischer Scientific) and a mouse anti-PICK1 antibody (2G10; described in (Jansen et al., 2009), 1:1000) for 1 h at room temperature. Cells were washed in blocking buffer and incubated for 45 min with Alexa Fluor 568 goat anti-rabbit and Alexa Fluor 647 goat anti-mouse (both 1:500, Life Technologies).

### Statistical analysis

The dataset was examined for normality (Shapiro–Wilk and Kolmogorov–Smirnov normality test), outliers were removed by Grubbs outlier analysis, and all data are presented as mean ± SEM. Significance level was set to *p* < 0.05. GraphPad Prism was used for data analyses except for three-way ANOVAs, which were analyzed in SPSS. All statistical analysis run are reported in detail in Table 1.

**Table 1.** Statistical table

Figure	Data structure	Type of test	Sample size	Statistical data
1*A* Cocaine administration in activity boxes with no habituation (PICK1 KO and WT mice)	Normal distribution	Two-way ANOVA followed by Holm–Sidak multiple comparison	WT:0 =145 = 1110 = 1230 = 12KO:0 = 125 = 1110 = 1130 = 11	Interaction:*p* = 0.32, *F*_(3,87)_ = 1.88Treatment:*p* ≤ 0.0001, *F*_(3,87)_ = 17.5Genotype:*p* = 0.0002, *F*_(1,87)_ = 14.91Multiple comparison, Treatment, df = 87:WT (compared to saline):5: *p* = 0.35, *t* = 0.9310: *p* = 0.002, *t* = 3.4130: *p* ≤ 0.0001, *t* = 6.15KO (compared to saline):5: *p* = 0.81, *t* = 0.2410: *p* = 0.36, *t* = 1.2830: *p* = 0.003, *t* = 3.45Multiple comparison, Genotype, df = 87:Saline: *p* = 0.45, *t* = 0.765: *p* = 0.34, *t* = 1.3210: *p* = 0.03, *t* = 2.5930: *p* = 0.02, *t* = 2.97
1*B–D*Cocaine administration in open field after a 120-min habituation (PICK1 KO and WT mice); drug comparison of 60 min before and after administration	Normal distribution	Three-way ANOVA of injection (last 60 min of habituation vs first 60 min of drug primed), genotype (WT vs KO), andtreatment (0- vs 10- vs 30-mg/kg cocaine)followed by *t* test(SPSS statistics)	WT:0 = 510 = 930 = 8KO:0 = 510 = 1030 = 10	Injection: *p* < 0.0001, *F* = 131.69, df = 1Genotype: *p* = 0.048, *F* = 4.16, df = 1Treatment: *p* < 0.0001, *F* = 14.24, df = 2Genotype*treatment: *p* = 0.21, *F* = 1.62, df = 2*t* test:Genotype effect after drug administration:Saline: *p* = 0.732, *t* = -0.355, df = 810: *p* = 0.01, *t* = 2.87, df = 1730: *p* = 0.44, *t* = 0.821, df = 16
2*A* Sensitization overview	NA	NA	NA	NA
2*B* Cocaine sensitization of WT mice	Normal distribution	Three-way ANOVA(SPSS statistics)	Saline:*n* = 9Cocaine:*n* = 11	Sensitization (day 1 vs 6)Day: *p* = 0.04, *F* = 4.61, df= 1Day*genotype: *p* = 0.72, *F* = 0.13, df = 1Day*treatment: *p* < 0.0001, *F* = 28.73, df = 1Day*genotype*treatment: *p* = 0.79, *F* = 0.07, df = 1Maintenance of sensitization (day 6 vs 12 vs 20)Day: *p* = 0.01, *F* = 5.13, df = 2Day*genotype: *p* = 0.46, *F* = 5.13, df = 2Day*treatment: *p* = 0.01, *F* = 5.32, df = 2Day*genotype*treatment: *p* = 0.71, *F* = 0.35, df = 2
2*C* Self-administration with cocaine	Normal distribution	Two-way ANOVA	WT:*n* = 7KO:*n* = 9	Interaction:*p* = 0.61, *F*_(4,70)_ = 0.68Cocaine doses:*p* = 0.0009, *F*_(4,70)_ = 5.28Genotype:*p* = 0.04, *F*_(1,70)_ = 4.46
2*D* Self-administration with liquid food	Normal distribution	Two-way ANOVA	WT:*n* = 9KO:*n* = 10	Interaction:*p* = 0.87, *F*_(4,85)_ = 0.30Food concentration:*p* < 0.0001, *F*_(4,85)_ = 18.82Genotype:*p* = 0.43, *F*_(1,85)_ = 0.63
3*A–C* SKF administration in open field after a 120-min habituation (PICK1 KO and WT mice); drug comparison of 60 min before and after administration	Normal distribution	Three-way ANOVA of injection (last 60 min of habituation vs first 60 min of drug primed), genotype (WT vs KO), andtreatment (0- vs 0.1- vs 1-mg/kg cocaine)followed by *t* test(SPSS statistics)	WT:0 = 50.3 = 81 = 8KO:0 = 50.3 = 91 = 8	Injection: *p* < 0.0001, *F* = 341.98, df = 1Genotype: *p* = 0.22, *F* = 1.58, df = 1Treatment: *p* < 0.0001, *F* = 21.15, df = 2Genotype*treatment: *p* = 0.75, *F* = 0.29, df = 2
3*D–F* Quinpirole administration in open field after a 120-min habituation (PICK1 KO and WT mice); drug comparison of 60 min before and after administration	Normal distribution	Three-way ANOVA of injection (last 60 min of habituation vs first 60 min of drug primed), genotype (WT vs KO), andtreatment (0- vs 0.1- vs 10- mg/kg cocaine)followed by *t* test (SPSS statistics)	WT:0 = 60.1 = 910 = 10KO:0 = 70.1 = 1010 = 8	Injection: *p* < 0.0001, *F* = 170.29, df = 1Genotype: *p* = 0.81, *F* = 0.06, df = 1Treatment: *p* = 0.44, *F* = 0.83, df = 2Genotype*treatment: *p* = 0.45, *F* = 0.83, df = 2
3*G* Surface levels of D_1_R	Normal distribution	One-sample *t* test	WT:*n* = 4KO:*n* = 5	*p* = 0.13 (two tailed), *t* = 1.91, df = 4
3*H* Striatal CREB protein levels	Normal distribution	One-sample t test	WT:*n* = 8KO:*n* = 8	*p* = 0.61 (two tailed), *t* = 0.53, df = 7
3*I* Striatal p-CREB protein levels		One-sample t test	WT:*n* = 4KO:*n* = 4	*p* = 0.98 (two tailed), *t* = 0.03, df = 3
4*A* V_max_ of synaptosomal DA uptake in WT vs PICK1 KO mice	Assuming normality	Unpaired t test	WT:*n* = 3KO:*n* = 3	V_max_: *p* = 0.025 (two tailed), *t* = 3.48, df = 4K_m_: *p* = 0.23 (two tailed), *t* = 1.43, df = 4
4*B* Saturation curve of synaptosomal DA uptake in WT vs PICK1 KO mice	NA	NA	NA	NA
4*C* Surface levels of DAT	Assuming normality	One-sample *t* test	WT:*n* = 4KO:*n* = 4	*p* = 0.39 (two tailed), *t* = 1.01, df = 3
4*D* Sucrose gradient showing DAT distribution	Assuming normality	One-sample *t* test	WT:*n* = 3KO:*n* = 3	*p* = 0.36 (two tailed), *t* = 1.18, df = 2
4*E* V_max_ of synaptosomal DA uptake in WT vs DAT + Ala mice	Assuming normality	Unpaired *t* test	WT:*n* = 4KO:*n* = 4	V_max_: *p* = 0.03 (two tailed), *t* = 2.74, df = 6K_m_: *p* = 0.04 (two tailed), *t* = 2.67, df = 6
4*F* Saturation curve of synaptosomal DA uptake in WT vs DAT + Ala mice	NA	NA	NA	NA
4*G* Cocaine administration in activity boxes with no habituation (DAT + Ala and WT mice)	Normal distribution	Two-way ANOVA followed by Holm–Sidak multiple comparison	WT:0 =95 = 810 = 1030 = 11KO:0 = 125 = 1110 = 1130 = 10	Interaction:*p* = 0.48, *F*_(3,74)_ = 0.84Treatment:*p* ≤ 0.0001, *F*_(3,74)_ = 20.31Genotype:*p* = 0.99, *F*_(1,74)_ = 4.810e-005Multiple comparison, Treatment, df = 74:WT (compared to saline):5: *p* = 0.12, *t* = 1.5610: *p* = 0.003, *t* = 3.2530: *p* ≤ 0.0001, *t* = 5.97KO (compared to saline):5: *p* = 0.03, *t* = 2.510: *p* = 0.03, *t* = 2.4130: *p* < 0.0001, *t* = 4.91Multiple comparison, Genotype, df = 74:Saline: *p* = 0.76, *t* = 0.445: *p* = 0.76, *t* = 1.0410: *p* = 0.76, *t* = 0.6730: *p* = 0.76, *t* = 0.89
5*A* Striatal DA levels measured by HPLC analysis	Normal distribution	Unpaired *t* test	WT:*n* = 7KO:*n* = 7	*p* = 0.01, *t* = 3.02, df = 12
5*B* Peak amplitude of KCl-evoked DA release	Normal distribution	Unpaired *t* test	WT:*n* = 5KO:*n* = 7	*p* = 0.04, *t* = 2.35, df = 10
5*C* Trace of peak amplitude of KCl-evoked DA release	NA	NA	NA	NA
5*D* Striatal VMAT2 protein levels	Normal distribution	One-sample *t* test	WT:*n* = 6KO:*n* = 6	*p* = 0.54, *t* = 0.66, df = 5
5*E* Striatal TH protein levels	Normal distribution	One-sample *t* test	WT:*n* = 10KO:*n* = 10	*p* = 0.008, *t* = 3.38, df = 9
5*F* Striatal pTH protein levels	Assuming normality	One-sample *t* test	WT:*n* = 3KO:*n* = 3	*p* = 0.38, *t* = 1.12, df = 2
5*G* Midbrain TH protein levels	Normal distribution	One-sample *t* test	WT:*n* = 8KO:*n* = 8	*p* = 0.92, *t* = 0.1, df = 7
5*H* Midbrain pTH protein levels	Assuming normality	One-sample *t* test	WT:*n* = 3KO:*n* = 3	*p* = 0.46, *t* = 0.90, df = 2
5*I* Midbrain TH mRNA levels	Assuming normality	One-sample *t* test	WT:*n* = 3KO:*n* = 3	*p* = 0.89, *t* = 0.16, df = 2
6*A* Midbrain IHC staining of TH and PICK1	NA	NA	WT:*n* = 3KO:*n* = 1	NA
6*B* Fluorescence polarization assay of TH-PICK1 binding	NA	NA	Six technical replicates from two individual experiments	NA
6*C* Coimmunoprecipitation of TH and PICK1 in striatum and midbrain	NA	NA	Three in each group	NA
6*D* Staining of GFP + TH + PICK1 following PICK1 KD in rat dopaminergic neurons	NA	NA	WT:*n* = 40KO:*n* = 40from two dissections, three transductions	NA
6*E* TH levels following PICK1 KD in rat dopaminergic neurons	Normal distribution	Unpaired *t* test	WT:*n* = 40KO:*n* = 40From two dissections, three transductions	*p* = 0.009, *t* = 2.68, df = 78
6*F* PICK1 levels following PICK1 KD in rat dopaminergic neurons	Non-normal	Mann–Whitney test	WT:*n* = 40KO:*n* = 40from two dissections, three transductions	*p* < 0.0001

Data were compared between groups using unpaired *t* tests when a Gaussian distribution was observed. Otherwise, the unparametric Mann–Whitney test was used. When comparing two normalized groups, with wild type set to 1 (Western blotting), one-sample *t* test was used. For cocaine administration without habituation of animals, as well as the self-administration paradigm, we used a two-way ANOVA with genotype and dose as factors. *F* values are reported as *F* (degrees of freedom between groups, degrees of freedom within groups). Pairwise multiple comparison procedures were made using the Holm–Sidak method. For drug-induced locomotion with habituation of animals as well as the sensitization paradigm, we instead used a three-way ANOVA with genotype, injection (before vs after drug) and dose as factors.

## Results

### Attenuated locomotor response to cocaine in mice with deletion of PICK1

**Figure 1.**
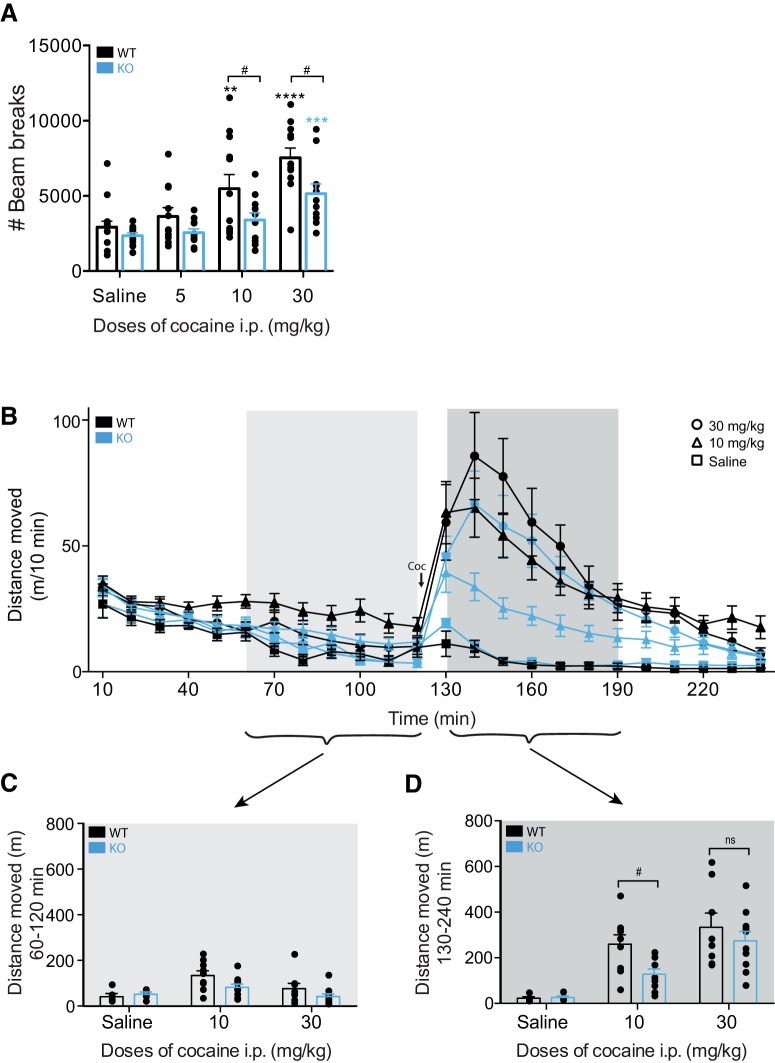
Impaired locomotor response to cocaine in PICK1 KO mice. ***A***, Mice were injected with the indicated doses of cocaine and locomotor activity (beam breaks) was recorded for 1 h. Two-way ANOVA followed by Holm–Sidak *post hoc* analysis revealed an overall significant difference between the genotypes (*F*_GENOTYPE(1,87)_ = 14.91, *p* = 0.0002, *F*_TREATMENT(3,87)_ = 17.5, *p* < 0.0001, *F*_INTERACTION(3,87)_ = 1.88, *p* = 0.32) with multiple comparison revealing genotype differences in the behavioral response to both 10- and 30-mg/kg cocaine (saline; *t*_(87)_ = 0.76, *p* = 0.45, 5 mg/kg; *t*_(87)_ = 1.32, *p* = 0.342, 10 mg/kg; *t*_(87)_ = 2.59, *p* = 0.033, 30 mg/kg; *t*_(87)_ = 2.97, *p* = 0.015). ***B–D***, Mice were placed in open field boxes and left to habituate for 120 min before being injected with the indicated doses of cocaine. Locomotor activity (distance moved in meters) was recorded for 2 h after cocaine injection. Analysis of the 60 min before and after drug injection reveals a significantly impaired cocaine response in the PICK1 KO mice (*F*_GENOTYPE_ = 4.16, df = 1, *p* = 0.048, *F*_TREATMENT_ = 14.24, *p* < 0.0001, df = 2, *F*_INJECTION_ = 131.69, *p* < 0.0001, df = 1, *F*_GENOTYPE*TREATMENT_ = 1.62, *p* = 0.21, df = 2). ***C***, Locomotor activity of the last 60 min before drug injection. ***D***, Locomotor activity of the first 60 min after drug injection with *post hoc* analysis revealing a significant difference at 10-mg/kg cocaine (saline; *t*_(8)_ = 0.36, *p* = 0.73, 10 mg/kg; *t*_(17)_ = 2.87, *p* = 0.01, 30 mg/kg; *t*_(16)_ = 0.82, *p* = 0.44). WT, wild type; * indicates versus own saline, # indicates genotype comparisons. All data expressed as mean ± SEM.

To investigate a possible role of PICK1 in mediating the behavioral actions of cocaine, we assessed cocaine-induced hyperactivity in PICK1 KO mice and littermate wild-type controls (Fig. 1). Mice were injected with 0-, 5-, 10-, or 30-mg/kg cocaine and placed directly in activity boxes for 1 h. Analysis of their locomotion revealed an overall treatment effect of cocaine with a significant difference between genotypes (*F*_GENOTYPE(1,87)_ = 14.91, *p* = 0.0002, *F*_TREATMENT(3,87)_ = 17.5, *p* < 0.0001, *F*_INTERACTION(3,87)_ = 1.88, *p* = 0.32; Fig. 1*A*). Further statistical assessment of the genotype effect revealed a significantly attenuated cocaine-induced behavioral response in the PICK1 KO mice after injection of both 10- and 30-mg/kg cocaine (saline; *t*_(87)_ = 0.76, *p* = 0.45, 5 mg/kg; *t*_(87)_ = 1.32, *p* = 0.342, 10 mg/kg; *t*_(87)_ = 2.59, *p* = 0.033, 30 mg/kg; *t*_(87)_ = 2.97, *p* = 0.015; Fig. 1*A*). Wild-type mice responded in a dose-dependent manner to 5-, 10-, and 30-mg/kg cocaine intraperitoneal by increased locomotor activity, while KO mice showed a significantly attenuated locomotor response with treatment effect only at the highest dose of cocaine (*F*_(3,87)_ = 17.5, *p* < 0.0001, wild type: 5 mg/kg; *t*_(87)_ = 0.93, *p* = 0.35, 10 mg/kg; *t*_(87)_ = 3.41, *p* = 0.002, 30 mg/kg; *t*_(87)_ = 6.15, *p* < 0.0001, KO: 5 mg/kg; *t*_(87)_ = 0.24, *p* = 0.81, 10 mg/kg; *t*_(87)_ = 1.28, *p* = 0.36, 30 mg/kg; *t*_(87)_ = 3.45, *p* = 0.003; Fig. 1*A*).

We then used an open field test to investigate cocaine-induced hyperactivity following a 120-min habituation period (Fig. 1*B–D*). Three-way ANOVA comparing injection (activity 60 min before vs after drug injection), genotype (wild type vs KO) and treatment (0- vs 10- vs 30-mg/kg cocaine), revealed significant effects of genotype, treatment and injection (pre vs post; *F*_GENOTYPE_ = 4.16, df = 1, *p* = 0.048, *F*_TREATMENT_ = 14.24, *p* < 0.0001, df = 2, *F*_INJECTION_ = 131.69, *p* < 0.0001, df = 1, *F*_GENOTYPE*TREATMENT_ = 1.62, *p* = 0.21, df = 2; Fig. 1*B*) with *post hoc* analysis revealing a significant difference at 10-mg/kg cocaine (saline; *t*_(8)_ = 0.36, *p* = 0.73, 10 mg/kg; *t*_(17)_ = 2.87, *p* = 0.01, 30 mg/kg; *t*_(16)_ = 0.82, *p* = 0.44; Fig. 1*C*) supporting the observation that the attenuated cocaine-induced locomotor response in PICK1 KO mice is present both with and without habituation (Fig. 1*A*).

### Decreased number of self-administered cocaine reinforcements in PICK1 KO mice

**Figure 2.**
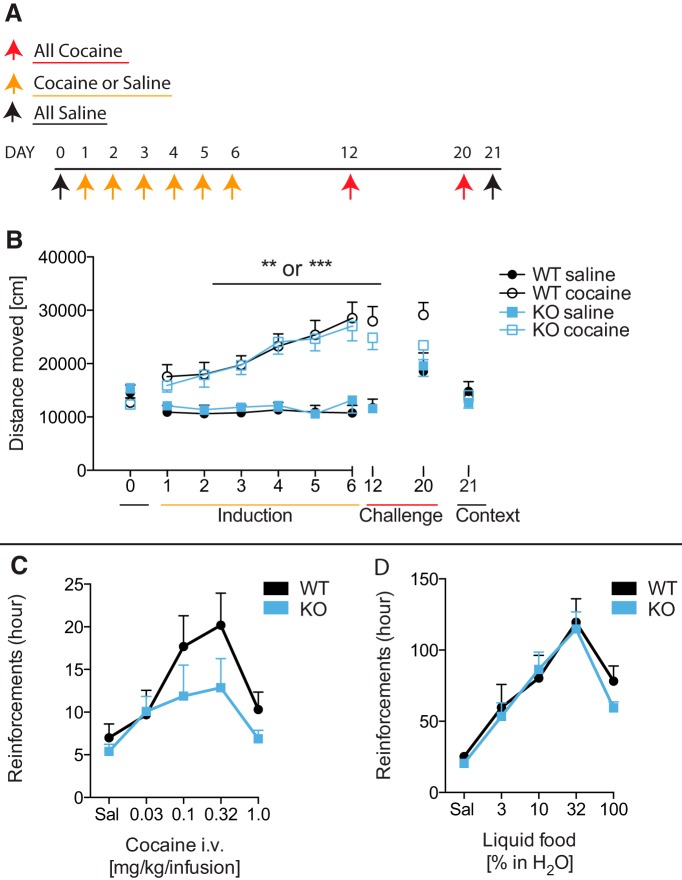
Maintained cocaine sensitization but fewer cocaine reinforcements in PICK1 KO mice. ***A***, Overview of the cocaine sensitization paradigm. ***B***, Cocaine sensitization of wild-type and PICK1 KO mice given daily doses of 0- or 10-mg/kg cocaine for 6 d followed by two challenges were all mice were given 10-mg/kg cocaine. Cocaine induced increased locomotion with no overall genotype difference (*F*_DAY1versus6_ = 4.61, *p* = 0.04, df = 1, *F*_DAY*GENOTYPE_ = 0.13, *p* = 0.72, df = 1, *F*_DAY*TREATMENT_ = 28.73, *p* < 0.0001, df = 1, *F*_DAY*GENOTYPE*TREATMENT_ = 0.07, *p* = 0.79, df = 1) and no difference in sensitization maintenance (*F*_DAY6versus12vs20_ = 5.13, *p* = 0.01, df = 2, *F*_DAY*GENOTYPE_ = 5.13, *p* = 0.46, df = 2, *F*_DAY*TREATMENT_ = 5.32, *p* = 0.01, df = 2, *F*_DAY*GENOTYPE*TREATMENT_ = 0.35, *p* = 0.71, df = 2). ***C***, Operant responding for cocaine in PICK1 KO and wild-type mice. PICK1 KO mice obtained significantly fewer cocaine reinforcements compared to wild-type mice in cocaine-maintained responding under a FR1 schedule of cocaine self-administration (*F*_INTERACTION(4,70)_ = 0.68, *p* = 0.61, *F*_COCAINEDOSE(4,70)_ = 5.28, *p* = 0.0009, *F*_GENOTYPE(1,70)_ = 4.46, *p* = 0.04). ***D***, Operant responding for liquid food in PICK1 KO and wild-type mice. Food-maintained operant behavior under the FR 1 schedule of reinforcement did not differ between PICK1 KO and wild-type mice (*F*_INTERACTION(4,85)_ = 0.30, *p* = 0.87, *F*_FOODDOSE(4,85)_ = 18.82, *p* < 0.0001, *F*_GENOTYPE(1,85)_ = 0.63, *p* = 0.43). All data expressed as mean ± SEM.

We next assessed whether the lack of PICK1 affected long-term behavioral effects of cocaine by testing PICK1 KO mice in a sensitization paradigm (Fig. 2). Mice were given daily injections of 10-mg/kg cocaine for 6 d followed by cocaine challenges on day 12 and day 20 (Fig. 2*A*). Control groups were given saline on the six induction days and cocaine on challenge days. Cocaine instigated increased cocaine-induced locomotor activity with progressively increasing response to cocaine in both wild-type and PICK1 KO mice with significant sensitization and no genotype difference (*F*_DAY1versus6_ = 4.61, *p* = 0.04, df = 1, *F*_DAY*GENOTYPE_ = 0.13, *p* = 0.72, df = 1, *F*_DAY*TREATMENT_ = 28.73, *p* < 0.0001, df = 1, *F*_DAY*GENOTYPE*TREATMENT_ = 0.07, *p* = 0.79, df = 1; Fig. 2*B*). In addition, there was no genotype difference in the maintenance of sensitization (*F*_DAY6versus12vs20_ = 5.13, *p* = 0.01, df = 2, *F*_DAY*GENOTYPE_ = 5.13, *p* = 0.46, df = 2, *F*_DAY*TREATMENT_ = 5.32, *p* = 0.01, df = 2, *F*_DAY*GENOTYPE*TREATMENT_ = 0.35, *p* = 0.71, df = 2; Fig. 2*B*).

We further explored whether lack of PICK1 affected cocaine’s reinforcing properties by testing PICK1 KO mice in an intravenous cocaine self-administration paradigm. PICK1 KO mice learned to self-administer cocaine as fast as their wild-type counterparts (Table 2), i.e., both genotypes acquired the task with similar number of reinforcers earned as well as days spent to reach criteria for baseline, extinction, and re-baseline (Table 2). However, when subsequently examining the dose-effect relationship (saline, 0.03, 0.1, 0.3, 1.0 mg/kg per infusion of cocaine), KO mice obtained significantly fewer cocaine reinforcements (*F*_INTERACTION(4,70)_ = 0.68, *p* = 0.61, *F*_COCAINE DOSE(4,70)_ = 5.28, *p* = 0.0009, *F*_GENOTYPE(1,70)_ = 4.46, *p* = 0.04; Fig. 2*C*) indicating an effect of lacking PICK1 on long-term effects of cocaine. A group of naïve mice was used for the control experiment with liquid food self-administration. While PICK1 KO mice were slightly slower to learn this task and earned fewer reinforcements during this phase of training, no differences were seen between genotypes during extinction and re-baseline criteria (Table 2), or during assessment of the dose-effect relationship (*F*_INTERACTION(4,85)_ = 0.30, *p* = 0.87, *F*_FOOD DOSE(4,85)_ = 18.82, *p* < 0.0001, *F*_GENOTYPE(1,85)_ = 0.63, *p* = 0.43; Fig. 2*D*), which indicates that the deficit in the self-administration paradigm primarily is related to cocaine. We should note, however, that mice were food restricted during the liquid food self-administration experiments, which, according to previous investigations, might affect DA dynamics (Roseberry, 2015; Jones et al., 2017).

**Table 2.** Mean number of days/reinforcers to reach criteria

	Days		RNF	
	WT	KO	WT	KO
Cocaine FR1 baseline	4.1 ± 0.6	3.9 ± 0.6	31.4 ± 4.1	25.6 ± 1.8
Cocaine FR1 extinction	2.6 ± 0.4	2.1 ± 0.2	12.1 ± 1.7	12.3 ± 1.8
Cocaine FR1 re-baseline	2.3 ± 0.6	2.4 ± 0.5	34.7 ± 4.3	42.2 ± 9.0
Liquid food FR1 baseline	2.9 ± 0.3	5.0 ± 0.7*	118.1 ± 5.8	92.1 ± 9.0*
Liquid food FR1 extinction	1.6 ± 0.2	1.3 ± 0.2	49.6 ± 5.9	42.6 ± 5.8
Liquid food FR1 re-baseline	2.9 ± 1.1	1.5 ± 0.3	156.3 ± 21.3	118.8 ± 8.6

Mice were trained with either cocaine or liquid food under FR1 schedule of reinforcement. PICK1 KO mice showed significantly lower liquid food reinforcements at FR1 acquisition criteria which was also reached slower than wild-type mice; unpaired *t* test; data are group mean ± SEM. Cocaine: *n*WT = 8 and *n*KO = 8; liquid food: *n*WT = 9 and *n*KO = 10.

### Preserved DA receptor activation and signal transduction in PICK1 KO mice

**Figure 3.**
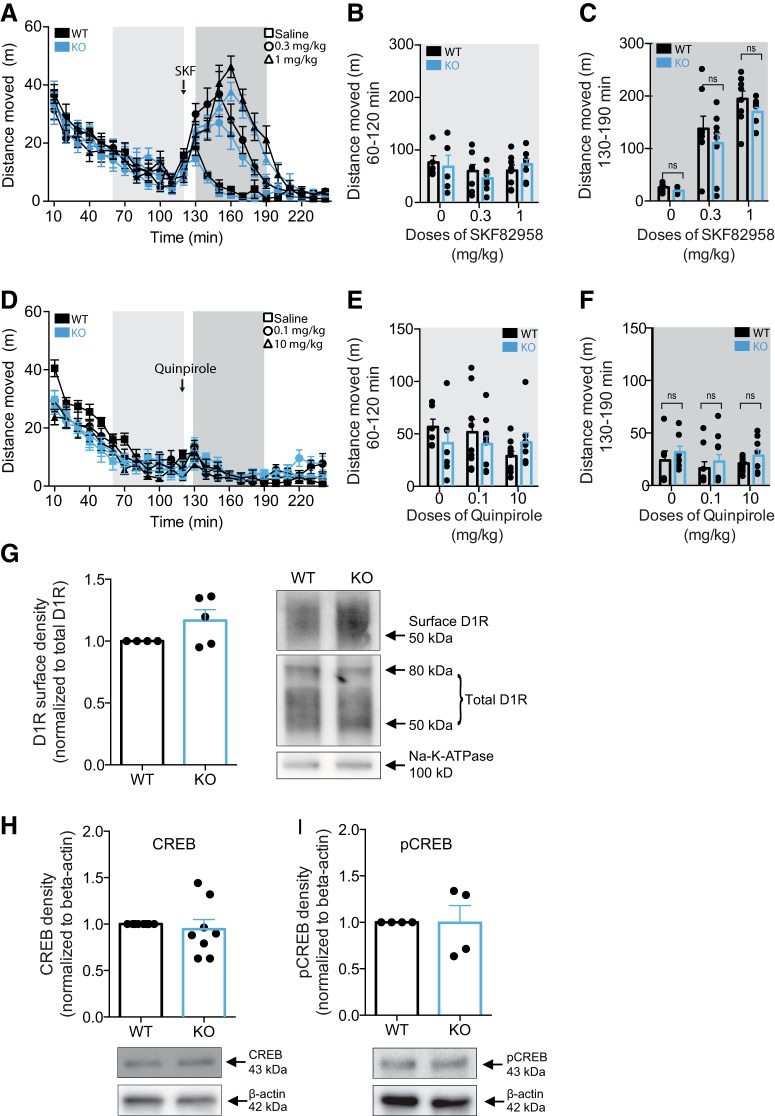
Preserved postsynaptic DA D_1_R signaling in PICK1 KO mice. ***A–C***, Mice were placed in open field boxes and left to habituate for 120 min before being injected with the indicated doses of SKF82958. Locomotor activity (distance moved in m) was recorded for 2 h after drug injection. Analysis of the 60 min before and after drug injection reveals a treatment effect, with no genotype difference on genotype response (*F*_GENOTYPE_ = 1.58, df = 1, *p* = 0.22, *F*_TREATMENT_ = 21.15, *p* < 0.0001, df = 2, *F*_GENOTYPE*TREATMENT_ = 0.29, *p* = 0.75, df = 2). ***B***, Locomotor activity of the last 60 min before drug injection. ***C***, Locomotor activity of the first 60 min after drug injection. ***D–F***, Mice were placed in open field boxes and left to habituate for 120 min before being injected with the indicated doses of quinpirole. Locomotor activity (distance moved in m) was recorded for 2 h after drug injection. Analysis of the 60 min before and after drug injection reveals no genotype difference on the behavioral effects of quinpirole (*F*_GENOTYPE_ = 0.06, df = 1, *p* = 0.81, *F*_TREATMENT_ = 0.83, *p* = 0.44, df = 2, *F*_GENOTYPE*TREATMENT_ = 0.83, *p* = 0.45, df = 2). ***E***, Locomotor activity of the last 60 min before drug injection. ***F***, Locomotor activity of the first 60 min after drug injection. ***G***, Immunoblotting of striatal lysates demonstrates that surface levels of D_1_R is unchanged in PICK1 KO mice (*t*_(4)_ = 1.91, *p* = 0.13). Left panel, Densitometric analysis of immunoblot for wild-type and PICK1 KO mice. Right panel, Representative immunoblot for D_1_R and Na-K-ATPase in wild-type and PICK1 KO mice. ***H***, ***I***, Immunoblotting of striatal lysates show unaltered expression levels of CREB and pCREB in PICK1 KO mice compared to wild-type controls (CREB: *t*_(7)_ = 0.53, *p* = 0.61, pCREB: *t*_(3)_ = 0.03, *p* = 0.98). Upper panels, Densitometric analysis of immunoblots for CREB and pCREB in wild-type and PICK1 KO mice. Lower panels, Representative immunoblots for pCREB and β-actin in wild-type and PICK1 KO mice. All data expressed as mean ± SEM.

To investigate whether the attenuated locomotor response to cocaine might result from aberrant postsynaptic D_1_R signaling in PICK1 KO mice, we challenged mice with the selective D_1_R agonist SKF82958 in the open field paradigm (Fig. 3*A–C*). Stimulation of DA D_1_R by SKF82958 has previously been shown to promote hyperlocomotor activity in mice and activation of downstream effectors similar to the effects of cocaine (Jeon et al., 2010; Beaulieu and Gainetdinov, 2011). DA D_1_R agonist stimulation induced significantly increased locomotor activity without genotype difference (*F*_GENOTYPE_ = 1.58, df = 1, *p* = 0.22, *F*_TREATMENT_ = 21.15, *p* < 0.0001, df = 2, *F*_GENOTYPE*TREATMENT_ = 0.29, *p* = 0.75, df = 2; Fig. 3*A–C*). We also tested a D_2_-receptor agonist quinpirole in a similar paradigm. Mice of both genotypes were allowed to habituate for 120 min followed by administration of quinpirole and assessment of locomotor activity. The treatment produced no significant difference between genotypes (*F*_INJECTION_ = 170, df = 1, *p* < 0.001, *F*_GENOTYPE_ = 0.06, df = 1, *p* = 0.81, *F*_TREATMENT_ = 0.83, *p* = 0.44, df = 2, *F*_GENOTYPE*TREATMENT_ = 0.83, *p* = 0.45, df = 2; Fig. 3*D–F*).

To investigate potential postsynaptic biochemical alterations in these mice, we probed the level of surface-expressed D_1_R using surface biotinylation in striatal slices (Fig. 3*G*). D_1_R surface expression was unaltered in PICK1 KO mice compared to wild-type mice (*t*_(4)_ = 1.91, *p* = 0.13; Fig. 3*G*). Furthermore, we prepared striatal lysates from wild-type and PICK1 KO mice and investigated expression and phosphorylation levels of cAMP response element binding (CREB) (Fig. 3*H*,*I*), a downstream effector of dopaminergic signaling (Beaulieu and Gainetdinov, 2011). Immunoblotting of striatal lysates showed that CREB levels in PICK1 KO mice (*t*_(7)_ = 0.53, *p =* 0.61; Fig. 3*H*) as well as levels of CREB pSer133 (*t*_(3)_ = 0.03, *p =* 0.98; Fig. 3*I*) were unaltered. Together, these data suggest that reduced behavioral responses of PICK1 KO mice to cocaine are unlikely due to compromised postsynaptic dopaminergic neurotransmission.

### PICK1 KO mice display reduced DA re-uptake despite preserved DAT surface expression

**Figure 4.**
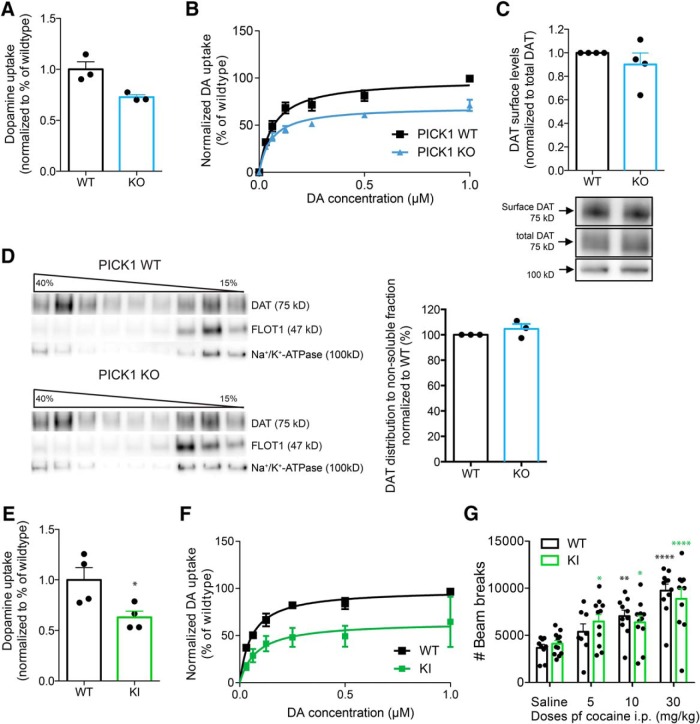
PICK1 KO mice show unaltered cellular distribution of DAT but reduced striatal DA uptake. DAT + Ala knock-in mice with a disrupted PDZ-binding motif demonstrate similar loss of DA uptake, albeit preserved locomotor response to cocaine. ***A***, ***B***, DA uptake in striatal synaptosomes from PICK1 KO mice and wild-type controls. Reduced DA uptake in striatal preparations from PICK1 KO mice (V_max_ 72.7 ± 24% of wild type; *t* = 3.48, *p* = 0.03, df = 4; ***A***). ***B***, Normalized saturation curve for DA uptake in striatal synaptosomes from PICK KO mice and wild-type littermate controls (maximal uptake capacity V_max_: wild type = 57.6 ± 4.3 fmol/min/μg; KO = 41.9 ± 1.4 fmol/min/μg, mean ± SEM). ***C***, Surface biotinylation of striatal slices demonstrate that PICK1 KO mice have preserved surface-expressed DAT compared to wild-type (*t*_(3)_ = 1.01, *p* = 0.39). Left panel, Densitometric analysis of immunoblots from wild-type and PICK1 KO mice. Right panel, Representative immunoblots for DAT and Na-K-ATPase. ***D***, Sucrose gradient centrifugation using striatal preparations show unaltered membrane distribution between the fractions with high and low buoyancy in PICK1 KO mice when compared to wild type. Left panel, Western blotting of a representative sucrose gradient fractionation from striatal lysates in a gradient from 15% to 40% and immunoblots for DAT, flottilin-1 (a marker of membrane rafts), and Na-K-ATPase. Right panel, Densitometric analysis showing the fraction of DAT distribution to nonsoluble (high buoyancy) fractions compared to wild-type controls (*t*_(2)_ = 1.18, *p* = 0.36). ***E***, DA uptake in striatal synaptosomes from DAT + Ala mice and wild-type controls. Reduced DA uptake in striatal preparations from DAT + Ala (V_max_ 63 ± 6% of wild type, *t* = 2.74, *p* = 0.03). ***F***, Normalized saturation curve for DA uptake in striatal synaptosomes from DAT + Ala mice and wild-type littermate controls (maximal uptake capacity V_max_: wild type = 49.87 ± 6.0 fmol/min/μg; KO = 31.36 ± 3.1 fmol/min/μg, mean ± SEM). ***G***, Assessment of cocaine-induced locomotor hyperactivity shows no genotype difference (*F*_INTERACTION(3,74)_ = 0.84, *p* = 0.48, *F*_TREATMENT(3,74)_ = 20.31, *p* < 0.0001, *F*_GENOTYPE(1,74)_ = 4.810e-005, *p* = 0.99). Black and green bars represent wild-type and corresponding DAT + Ala mice. There was no treatment difference between treatment groups. All data expressed as mean ± SEM.

We have previously reported that the expression and distribution of DAT in striatum of PICK1 KO mice are unaffected compared to wild-type mice (Rickhag et al., 2013). Here, we investigated whether the attenuated behavioral effects of cocaine (Figs. 1, 2) could be related to compromised presynaptic DAT function. We therefore assessed functional levels of DAT in striatum by evaluating [^3^H]-DA uptake in synaptosomal preparations. Interestingly, we found significantly reduced DA uptake capacity in PICK1 KO mice compared to wild-type controls (V_max_ 72.7 ± 24% of wild type; *t* = 3.48, *p* = 0.03, df = 4; Fig. 4*A*) without a change in DA affinity (K_m_ for wild type: 84 ± 23 nM; K_m_ for KO: 50 ± 5 nM, *t* = 1.43, *p* = 0.23, df = 4). Levels of surface expressed DAT, however, was not significantly reduced as determined by surface biotinylation experiments on striatal slices (*t*_(3)_ = 1.01, *p =* 0.39; Fig. 4*C*). We were also unable to detect any change in microdomain compartmentalization of DAT in the plasma membrane of striatal slices (Fig. 4*D*). Indeed, previous data have supported that DAT is sequestered into cholesterol-enriched microdomains or membrane rafts in the plasma membrane (Adkins et al., 2007; Cremona et al., 2011). According to sucrose gradient fractionation experiments, DAT was to a similar degree segregated into the detergent-resistant (membrane raft) fractions, together with the membrane raft marker flotilin 1 (Flot1), in lysates from PICK1 KO mice compared to wild-type lysates (*t*_(2)_ = 1.18, *p* = 0.36; Fig. 4*D*). Together, the data demonstrate that decreased DA uptake in PICK1 KO mice cannot be explained by decreased DAT expression and membrane distribution in striatum.

### Compromised DA re-uptake but preserved locomotor response to cocaine in DAT knock-in mice with disrupted PDZ-binding motif

We have previously generated knock-in mice expressing DAT mutants incapable of binding PDZ-domain proteins such as PICK1 (Rickhag et al., 2013). These mice show reduced striatal DAT expression, but given the unchanged expression and distribution of DAT in PICK1 KO mice, the reduced striatal DAT expression in these mice was unlikely the result of disrupted PICK1 binding (Fig. 4*A–C*; Rickhag et al., 2013). Interestingly, however, in DAT + Ala mice, we found that the [^3^H]-DA uptake capacity was significantly reduced as seen in PICK1 KO mice. The measured DA uptake in synaptosomes from DAT + Ala mice was ∼63% of wild-type (DAT + Ala: V_max_ 63 ± 6% of wild type, *t* = 2.74, *p* = 0.03; Fig. 4*E*,*F*) compared to ∼73% for PICK1 KO mice (Fig. 4*A*,*B*). Nonetheless, the DAT + Ala mice displayed unchanged cocaine-induced hyperactivity compared to wild-type littermates at all of the tested cocaine doses (5, 10, and 30 mg/kg, i.p.; *F*_INTERACTION(3,74)_ = 0.84, *p* = 0.48, *F*_TREATMENT(3,74)_ = 20.31, *p* < 0.0001, *F*_GENOTYPE(1,74)_ = 4.810e-005, *p* = 0.99; Fig. 4*G*). These data suggest that the attenuated behavioral response to cocaine in PICK1 KO mice is unrelated to PICK1-DAT interactions, or to the 20–30% reduction in DAT reuptake capacity in PICK1 KO mice.

### Evidence for altered DA homeostasis in PICK1 KO mice

**Figure 5.**
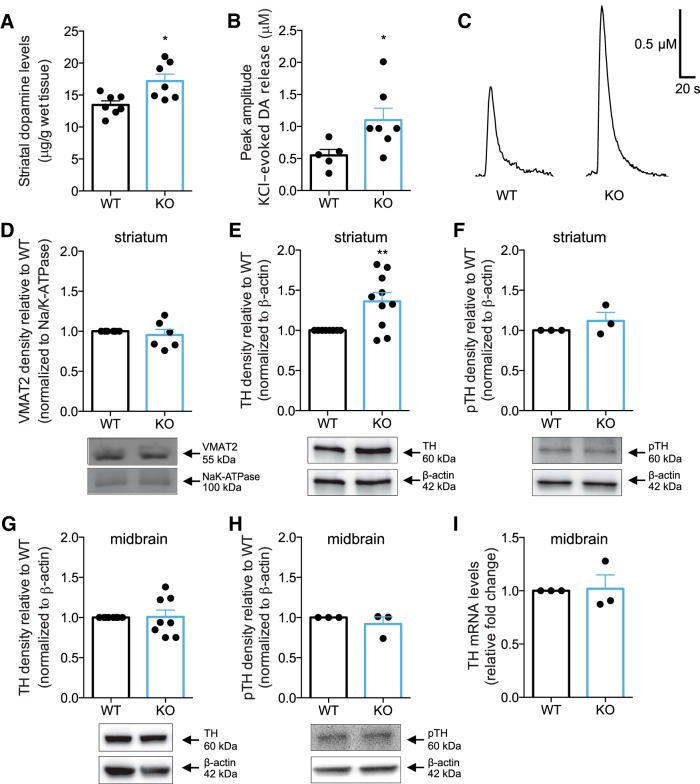
Increased TH protein levels and DA content in striatum, correlates with enhanced DA release in PICK1 KO mice. ***A***, Total DA content in striatal homogenates from PICK1 KO mice and wild-type controls as measured by HPLC. PICK1 KO mice show significantly elevated DA content in striatum (∼128% of wild type, *t*_(12)_ = 3.02, *p* = 0.01). ***B***, ***C***, Enhanced vesicular DA release in striatum of PICK1 KO mice as measured by high-speed *in vivo* chronoamperometry. Data show peak amplitude on KCl-evoked DA release in striatum (*t*_(10)_ = 2.35, *p* = 0.04). The same amount of KCl pressure-ejected locally into striatum elicited 2-fold greater DA release in PICK1 KO mice relative to wild-type mice. Shown are representative oxidation currents, converted to a micromolar concentration using a calibration factor determined for each electrode *in vitro*. ***D***, Immunoblotting from striatal lysates shows unchanged VMAT2 expression in PICK1 KO mice (*t*_(5)_ = 0.66, *p =* 0.54). ***E***, Immunoblotting revealed elevated TH protein expression in striatal lysates from PICK1 KO mice (*t*_(9)_ = 3.38, *p* = 0.008). Upper panels, Densitometric analysis of immunoblots for wild-type and PICK1 KO mice. Lower panels, Representative immunoblots for TH and β-actin in wild-type and PICK1 KO mice. ***F***, Phosphorylation of TH at serine-40 is unaltered in striatum of PICK1 KO mice (*t*_(2)_ = 1.12, *p* = 0.38). Upper panels, Densitometric analysis of immunoblots for wild-type and PICK1 KO mice. Lower panels, Representative immunoblots for pTH and β-actin in wild-type and PICK1 KO mice. ***G***, Immunoblotting from ventral midbrain lysates show unchanged TH expression in PICK1 KO mice (*t*_(7)_ = 0.1, *p* = 0.92). ***H***, Immunoblotting from ventral midbrain lysates show unchanged pTH expression in PICK1 KO mice (*t*_(2)_ = 0.90, *p* = 0.46). ***I***, Quantitative PCR analysis show unaltered TH mRNA levels in midbrain of PICK1 KO mice compared to wild-type control (*t*_(2)_ = 0.16, *p* = 0.89). All data expressed as mean ± SEM.

To correlate putative dysregulation of DA homeostasis with altered behavioral responses in PICK1 KO mice, we tested markers for DA synthesis, DA reuptake and vesicular storage together with DA release kinetics from intact striatal terminals. Strikingly, striatal DA content was significantly elevated in PICK1 KO mice as measured by high-performance liquid chromatography analysis of striatal lysates (*t*_(12)_ = 3.02, *p* = 0.01; Fig. 5*A*). To investigate if increased striatal pools of DA were associated with functional consequences *in vivo*, we used high-speed chronoamperometric recordings in striatum to measure DA release in PICK1 KO and littermate wild-type mice. KCl was pressure-ejected into striatum of anaesthetized mice to evoke DA release. In agreement with increased striatal DA content, KCl pressure-ejection locally into striatum elicited two-fold greater DA release in PICK1 KO mice relative to wild-type mice (*t*_(10)_ = 2.35, *p* = 0.04; peak amplitude for wild type: 0.61 μM; KO: 1.24 μM; Fig. 5*B*). Representative traces of DA release in mouse striatum on KCl stimulation are shown in Figure 5*C*. Clearance rate (wild type: 46 ± 11 nM/s; KO: 72 ± 26 nM/s) was not significantly different between genotypes; however, we should note that it is difficult to interpret precisely these rates given that KCl-induced DA release likely continues simultaneously with clearance, especially during the initial phase of the descending limb of the signal. Nonetheless, in sum, elevated evoked DA release in PICK1 KO mice supports our finding of increased DA content in striatum.

Vesicular monoamine transporter 2(VMAT2) plays an essential role in DA homeostasis by sequestering DA into synaptic vesicles. However, VMAT2 expression in striatal preparations was not significantly different between genotypes (*t*_(5)_ = 0.66, *p =* 0.54; Fig. 5*D*), implying that elevated DA in striatum is not associated with increased VMAT2 expression.

Immunoblotting showed that levels of the rate-limiting enzyme of DA synthesis, TH, were significantly increased in striatal terminals from PICK1 KO mice (*t*_(9)_ = 3.38, *p =* 0.008; Fig. 5*E*). Since TH phosphorylation at Ser40 by protein kinase A positively regulates catalytic activity of TH (Lindgren et al., 2000), we determined TH phosphorylation at Ser40. The relative phosphorylation of this site, however, was not different between genotypes (*t*_(2)_ = 1.12, *p* = 0.38; Fig. 5*F*). We also determined TH levels in the ventral midbrain from where the mesostriatal DA projections originate, and thus, in the somatodendritic compartment of dopaminergic neurons (Björklund and Dunnett, 2007). In ventral midbrain lysates, levels of TH were not different between the genotypes (*t*_(7)_ = 0.1, *p* = 0.92; Fig. 5*G*) suggesting differential regulation of TH in neuronal perikarya compared to axonal terminals in striatum. In addition, we observed no difference in the relative phosphorylation of Ser40 in TH (*t*_(2)_ = 0.90*, p* = 0.46; Fig. 5*H*). Importantly, quantitative PCR analysis from midbrain preparations revealed no alterations in TH mRNA levels in the absence of PICK1, which eliminate the possibility that transcriptional changes can account for the phenotype (*t*_(2)_ = 0.16, *p* = 0.89; Fig. 5*I*).

### PICK1 is localized to TH-expressing neurons in ventral midbrain

**Figure 6.**
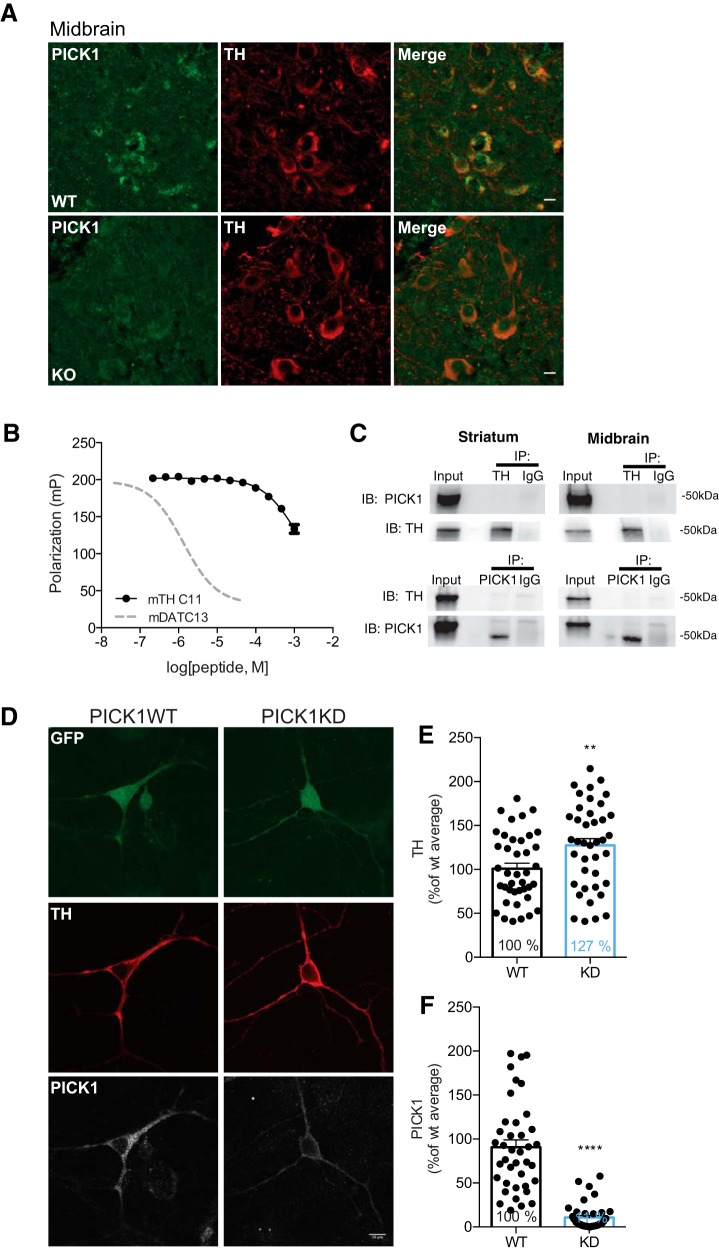
PICK1 is expressed in TH-positive midbrain neurons and lentiviral KD in midbrain dopaminergic cell cultures causes elevated TH expression. ***A***, Immunohistochemical analysis demonstrates PICK1 immunofluorescence in TH-expressing neurons in ventral midbrain. Neuronal cell bodies in ventral midbrain show diffuse TH distribution in the entire cytosol while PICK1 immunofluorescence is clustered and polarized in the neuronal cell bodies. Scale bar: 10 μm. ***B***, Competition fluorescence polarization indicates a non-PDZ-mediated interaction between TH and PICK1. Graph displays the competition of the OrgDAT with unlabelled TH-C11, corresponding to the 11 most C-terminal residues in TH or unlabeled DAT13 corresponding to the 13 most C-terminal residues in DAT (dotted line). ***C***, Coimmunoprecipitation experiments from both midbrain and striatal preparations show lack of any direct interaction between TH and PICK1. Left upper left panel, IP of TH from striatum shows no co-IP of PICK1 (*n* = 3). Right upper panel, IP of TH from midbrain shows no co-IP of PICK1 (*n* = 3). Left lower panel, IP of PICK1 from striatum shows no co-IP of TH (*n* = 1). Right lower panel, IP of PICK1 from midbrain shows no co-IP of TH (*n* = 1). ***D***, Representative confocal images of postnatal midbrain cultures of rat dopaminergic neurons, virus transduced with vectors containing the constructs for cytosolic GFP (left panel) or cytosolic GFP and the endogenous PICK1 silencing RNA sh18 (right panel). Scale bar: 10 μm. ***E***, Significantly elevated TH expression in PICK1 KD versus wild-type neurons (*t*_(78)_ = 2.68, *p =* 0.009; ∼127% compared to wild type). ***F***, KD efficiency of the lentiviral PICK1 KD show significant reduction of PICK1 (*p* < 0.0001). All data expressed as mean ± SEM.

To further explore whether PICK1 indirectly or directly, acts as a negative regulator of striatal TH levels without changing the balance between TH and phosphorylated TH, we performed immunofluorescence for PICK1 and TH in midbrain slices. Previous reports demonstrate immunolabeling of PICK1 both at pre- and postsynaptic sites in cortex, hippocampus, cerebellum and striatum (Haglerød et al., 2009, 2017). Here, we report using an optimized immunohistochemistry protocol with tyramide signal amplification, immunofluorescence of PICK1 in midbrain dopaminergic neurons as evidenced by colabeling with TH (Fig. 6*A*) supporting a previous study validating PICK1 expression in dopaminergic neurons (Torres et al., 2001). PICK1 expression was localized predominantly in the cytosolic compartment of TH-expressing neurons in a polarized fashion with a punctate pattern. The immunosignal of PICK1 showed clear overlap with the TH signal, which as expected (Apuschkin et al., 2015) was localized to the cytosol, although more diffuse in its distribution as compared to that of PICK1 (Fig. 6*A*). Importantly, no PICK1 signal was seen in slices from PICK1 KO mice.

With the binding promiscuity of the PICK1 PDZ-domain in mind (Erlendsson et al., 2014), we assessed whether TH could be a PICK1 PDZ-domain binding partner. We used a fluorescence polarization assay and measured the ability of a peptide corresponding to the 11 most C-terminal residues of mouse TH (mTH C11) to inhibit binding of purified PICK1 to a fluorescently tagged peptide corresponding to the most 13 C-terminal residues of the known PICK1 interaction partner DAT (OrgDATC13). Whereas the unlabeled DAT peptide (mDATC13) potently inhibited binding of OrgDATC13, the potency of mTHC11 was several orders of magnitude weaker (Fig. 6*B*). This excludes canonical high-affinity binding of TH to the PICK1 PDZ-domain but does not exclude a noncanonical binding mode between the two proteins as, for example, seen for the PICK1:Arf1 interaction (Takeya et al., 2000; Rocca et al., 2013; Erlendsson et al., 2014). We therefore also performed coimmunoprecipitation experiments on both midbrain and striatal lysates. However, in both lysates neither immunoprecipitation of PICK1 nor TH led to coprecipitation of TH or PICK1, respectively (Fig. 6*C*), further arguing against direct interaction between the two proteins.

### KD of PICK1 in midbrain dopaminergic neurons enhances TH expression

We hypothesized that KD of PICK1 specifically in DA neurons would result in elevated TH expression as observed in striatal lysates from PICK1 KO mice. Despite the lack of a direct PDZ-domain-mediated protein-protein interaction between TH and PICK1 (Fig. 6*B*), elevated TH and DA levels in PICK1 KO mice could indicate an indirect role for PICK1 in the regulation of TH expression and thereby DA synthesis. We used a dual promoter lentiviral vector to simultaneously KD PICK1 with shRNA under control of the human H1 promoter and eGFP under control of the ubiquitin promoter for identification of transduced neurons. Cells that were double positive for GFP and TH staining were imaged and identified as neurons of interest. Immunostaining showed widespread, diffuse eGFP distribution in transduced neurons (Fig. 6*D*, top panel), TH staining showed cytosolic localization (Fig. 6*D*, middle panel), while PICK1 expression was particularly punctate (Fig. 6*D*, lower panel) consistent with the brain slice imaging (Fig. 6*A*). Transduction with PICK1 shRNA resulted in an efficient PICK1 KD (Fig. 6*D*, lower right panel, *F*). Remarkably, the KD of PICK1 in dopaminergic neurons caused a significant increase in TH levels (*t*_(78)_ = 2.68, *p =* 0.009 (∼127% compared to wild type, control GFP vs ShPICK1 GFP; Fig. 6*D*, middle panel, *E*), which is in accordance with the increased TH found in the PICK1 KO mice.

## Discussion

PICK1 is known to interact via its PDZ-domain with a range of different membrane proteins and kinases (Xu and Xia, 2006; Li et al., 2016) including DAT, which is the main target for the stimulatory actions of cocaine (Giros et al., 1996). The significance of PICK1 for the actions of cocaine has nonetheless remained elusive. Our study demonstrates that PICK1 is critical for both acute and chronic behavioral actions of cocaine. In addition, our data reveal a striking role of PICK1 in regulating DA homeostasis. This regulation appears to be unrelated to PICK1’s interaction with DAT, but likely involves negative regulation of striatal TH expression, as reflected by increased levels of TH and DA in striatum, as well as enhanced DA release in PICK1 KO mice.

The widely abused psychostimulant cocaine inhibits DAT leading to a rise in extracellular DA, stimulation of DA receptors, and a dose-dependent hyperlocomotion (Giros and Caron, 1993; Giros et al., 1996). This increase in locomotor activity in response to cocaine was markedly attenuated in PICK1 KO mice (Fig. 1). In addition, PICK1 KO mice obtained significantly fewer cocaine reinforcements in an intravenous self-administration paradigm of cocaine (Fig. 2*C*). Importantly, cocaine self-administration (Kmiotek et al., 2012) assesses motivational aspects of cocaine seeking in contrast to sensitization (Post and Rose, 1976; Steketee and Kalivas 2011), which involves passive cocaine administration and only assesses locomotion. Overall, these data support critical involvement of PICK1 in both acute effects of and chronic, neuroadaptive behavioral changes induced by cocaine. A role of PICK1 in changes induced by cocaine has been indicated previously in studies showing that disruption of PICK1-GluA2 interactions abolish cocaine-induced synaptic plasticity in striatum and VTA (Bellone and Lüscher, 2006; Famous et al., 2008). Furthermore, inhibition of PICK1 in nucleus accumbens attenuates reinstatement of cocaine seeking (Famous et al., 2008; Schmidt et al., 2011).

Our data suggest that postsynaptic DA signaling is not important for the diminished behavioral effects of cocaine in PICK1 KO mice. When PICK1 KO mice were challenged with a D_1_R agonist, we observed dose-dependent increases in locomotor activity that did not differ from wild-type littermates (Fig. 3*A–C*). Additionally, we observed no behavioral difference between genotypes when challenging the mice with a D_2_R agonist (Fig. 3*D–F*), and striatal levels of CREB/pCREB were not different between genotypes (Fig. 3*H*,*I*). Our results also suggest that the attenuated behavioral effects of cocaine in PICK1 KO mice are unrelated to the interaction between PICK1 and DAT (Fig. 4*A–D*). Previous investigations based on a yeast two-hybrid screen and coimmunoprecipitation experiments suggested a direct interaction between DAT and PICK1 (Torres et al., 2001). Nonetheless, conflicting data exist regarding the influence of PICK1 on DAT function. Overexpression of PICK1 was found to enhance DAT surface expression and DA uptake in heterologous cell lines and immortalized dopaminergic neurons (Torres et al., 2001). However, later studies reported that PICK1 lacks influence on both DAT surface levels and DA uptake (Bjerggaard et al., 2004; Madsen et al., 2012) and we previously showed that total DAT protein expression is unchanged in PICK1 KO mice (Rickhag et al., 2013). Here, we find unaltered DAT surface expression in the PICK1 KO mice (Fig. 4*C*) concomitant with preserved microdomain distribution in striatum (Fig. 4*D*). In sum, these data suggest that PICK1 is not critical for overall DAT surface distribution in striatal terminals. Despite preserved DAT distribution, however, we observed a decrease in synaptosomal DA uptake in PICK1 KO mice (Fig. 4*A*,*B*).

We recently described DAT knock-in mice that express mutations hindering the interaction between the DAT C-terminus and PDZ-domain scaffold proteins. For the DAT + Ala mice, expressing a mutation with an extra alanine added to the C-terminus, we reported a marked reduction in striatal DAT protein expression (Rickhag et al., 2013). Here, we show that the reduction in protein expression is paralleled by a reduction in DA uptake (Fig. 4*E*,*F*). These observations contrast the PICK1 KO mice in which DAT expression and distribution are unchanged compared to wild-type mice despite similar reduction of DA uptake capacity. Thus, the reduction in DAT levels in DAT + Ala mice is conceivably a consequence of disrupting the interaction between DAT and other yet unknown PDZ-domain scaffolding proteins. Interestingly, despite the reduction in functional DAT levels, we observe no change in the acute locomotor response to cocaine in the DAT + Ala mice (Fig. 4*G*). This supports the notion that even rather large changes in DAT level do not necessarily affect the acute actions of cocaine. This finding is consistent with earlier studies using DAT heterozygous mice, which have a 50% reduction in DAT, but are behaviorally fully responsive to cocaine and amphetamine (Giros et al., 1996; Jones et al., 1998, 1999). Homozygous DAT KO mice, however, are behaviorally nonresponsive to cocaine, consistent with DAT being the primary target for cocaine (Giros et al., 1996; Jones et al., 1998, 1999). In summary, we argue that the ∼30% reduction in DAT function seen in PICK1 KO mice is unlikely to account for the attenuated behavioral response to cocaine in these mice.

To further explore mechanisms underlying the impaired cocaine responses in PICK1 KO mice, we investigated key parameters related to DA homeostasis. Levels of the rate-limiting enzyme in DA synthesis, TH, were significantly elevated in striatum of PICK1 KO mice (Fig. 5*E*), suggesting increased DA synthesis capacity in this region. There was no change, however, in midbrain TH levels and no transcriptional change, which suggests that a role of PICK1 in regulation of TH protein levels is selective to mesostriatal projections. Of interest, differential regulation of TH levels has been reported before; a differential reduction of TH was observed in DAT KO mice between the somatodendritic compartment and the striatal terminals (Jaber et al., 1999; Salvatore et al., 2016). The difference in TH levels between striatum and ventral midbrain in PICK1 KO mice could be related to either a trafficking dysfunction or differential regulation of TH in neuronal perikarya compared to striatal terminals. Despite unchanged levels of Ser40 phosphorylated TH (Lindgren et al., 2000), the increased striatal TH levels observed here are indicative of increased DA synthesis capacity and consistent with this, we found higher DA levels in striatal homogenates from PICK1 KO mice compared to wild-type littermates (Fig. 5*A*). Moreover, high-speed chronoamperometric recordings in striatum of PICK1 KO mice revealed increased DA release on KCl stimulation (Fig. 5*B*,*C*), consistent with increased striatal DA in PICK1 KO mice, which most likely can be attributed to increased synthesis and DA storage. Of interest, overexpression of the VMAT2 enhances vesicular storage capacity for DA and increases striatal tissue DA levels (Lohr et al., 2014). In PICK1 KO mice, however, VMAT2 expression was unaltered compared to wild type (Fig. 5*D*), and abnormal VMAT2 expression can therefore not account for increased striatal DA in these mice.

The ability of PICK1 to regulate TH levels is most likely inherent to dopaminergic neurons. Indeed, we observed PICK1 expression in TH-immunolabeled neurons in midbrain (Fig. 6*A*) in agreement with previous data (Torres et al., 2001). Furthermore, we found that lentiviral shRNA-mediated KD of PICK1 in isolated, cultured midbrain dopaminergic neurons enhances TH expression (Fig. 6*D–F*), providing strong evidence for a functional link between PICK1 and TH. Importantly, elevated TH expression following acute PICK1 KD in dissociated dopaminergic neurons, where neurons are disconnected from synaptic input, strongly argues that the observed TH increase in PICK1 KO mice is not explained by compensatory pre- or postsynaptic adaptations caused by the global KO of PICK1. Based on these findings, it was obvious to suspect a direct interaction between PICK1 and TH. However, the mouse TH C-terminus showed very poor affinity for the PICK1 PDZ-domain (Fig. 6*B*). Moreover, coimmunoprecipitation experiments did not support any association between PICK1 and TH (Fig. 6*C*). This suggests that the exact manner by which PICK1 regulates TH remains elusive. It might be speculated that this regulation depends on the interaction of PICK1 with one of its other binding partners, but future investigations are warranted to address this issue.

In conclusion, we show that deletion of PICK1 markedly impairs behavioral responses to cocaine. It is unlikely that the impaired acute locomotor response to cocaine is related to the interaction between PICK1 and DAT. This deficit might rather be attributed to significant changes in DA synthesis and release dynamics in striatal nerve terminals. Indeed, the higher levels of resting DA levels in concert with decreased DA uptake capacity implies that the window of resting and saturation levels is limited, which could be part of the explanation to the impaired behavioral response to cocaine. Notably, increased tissue levels of monoamines promoted by overexpression of *Drosophila* VMAT in fly brain impair behavioral responses to cocaine (Chang et al., 2006). We also observed reduced cocaine self-administration consistent with involvement of PICK1 in regulation of neuroadaptive changes occurring after repeated cocaine exposures. By establishing PICK1 as a key player in psychostimulants action, our data provide an important framework for exploring PICK1 as putative target for treatment of drug abuse.

## Synthesis

Reviewing Editor: Christian Lüscher, University of Geneva Decisions are customarily a result of the Reviewing Editor and the peer reviewers coming together and discussing their recommendations until a consensus is reached. When revisions are invited, a fact-based synthesis statement explaining their decision and outlining what is needed to prepare a revision will be listed below. The following reviewer(s) agreed to reveal their identity: Erin Calipari, Marc Exton-McGuinness

The ms is now much improved and no new experiments are needed. Howver I ask the authors to address the points regarding the analysis of the open field data as well as the DA uptake experiment, where a paird t test cannot be used. Here are the referees comments

Major points:

I apologise if I did not convey my opinion on the open field analysis clearly in my previous comments. By 'within-session' my intention was to convey that it is more appropriate to directly compare habituation with the drug-loaded state. Essentially this means performing a 3-way ANOVA, with Genotype [wt vs KO], Treatment [saline vs 10 vs 30], and Injection [habituation vs. drug-primed]. This will statistically confirm that the drug was effective at increasing activity (or not in the case of quinpirole) which is not captured by the current analysis. However, I would highlight that the habituation and drug-loaded bins must contain an equal number of minutes (otherwise the analysis will be unbalanced and invalid).

On a minor related point, I am not sure I accept the authors' argument for excluding the first 10 mins post-injection as removing 'experimenter-induced locomotion' (line 152), unless there was some artefact with the recording software. There doesn't appear to be any evidence of an experimenter-induced artefact in the data figures? This also has the added effect of unbalancing the habituation and drug-loaded analyses. While responding does increase in saline controls when reintroduced to the open-field, this is an entirely natural and expected exploration response.

However, I would highlight that it is perfectly acceptable (and common practise) to analyse shorter time periods - for example the final 30mins of habituation vs the first 30mins post drug challenge. This will still answer the authors' research question but has the effect of excluding irrelevant periods of time, such as those at the end of the session where the drug has worn-off or those at the beginning of habituation in which locomotion is heavily novelty-driven. The main data can still be presented as is, however with minor alterations to emphasise which time periods were used for analysis (although Fig 1C and D would need to be edited to reflect the shorter analysis periods).

I believe that collapsing multiple bins to create larger 'habituation' and 'drug-loaded' sessions is the most appropriate way to approach this - although presenting the bins within the figure is still useful (and again common practise). At present the within-session analysis is not particularly informative as the sheer number of comparisons makes interpretation difficult even with corrections applied, moreover they do not compare habituation vs post-injection.

My final major point concerns the analysis of the DA uptake data. The methods states: “we used a paired t-test, since this has more power than the normal t-test and is applicable when the data set demonstrates extensive variation, which was the case”. I don't understand the basis for this statement, as the way this is written suggests this approach most certainly is not valid. “Extensive variation” is no justification for a paired test over an unpaired one, and heavy tailed distributions can be highly damaging to error rates for conventional parametric tests. It is not appropriate to pairwise compare KOs and WTs as observations from each are independent (i.e. from different synapses in different mice). While it would be appropriate to subtract a baseline from each observation of the same neuron/synapse, observations between KOs and WTs are independent samples.

Minor points:

1) Line 563: It does not seem appropriate to analyse the WT and KOs separately. To compare across these two a correction would be required. A 3 factorial Genotype x Drug x Day would be more appropriate. Ditto Line 575.

2) Line 590 (also 824): On reflection it is perhaps clearer to simply explicitly describe this observation as “KO mice obtained significantly fewer cocaine reinforcements”. Describing this as a 'blunting' has its difficulties as we don't know what is being blunted? This is an interesting question, although beyond the scope of the present discussion. One cannot determine in the present paradigm (indeed most paradigms) whether reductions in reinforcements are due to impairments in incentive or hedonic processing - or even impulsivity differences.

3) For DA uptake the comparison between WT and KO is presented as if it were significant; however, p=0.06 (and I question the validity of this statistical approach). While there is a decrease it should be made clearer that this observation is non-significant. Although this does not affect the final conclusions of this experiment.
